# Translating Biospectroscopy Techniques to Clinical Settings: A New Paradigm in Point-of-Care Screening and/or Diagnostics

**DOI:** 10.3390/jpm13101511

**Published:** 2023-10-19

**Authors:** Francis L. Martin

**Affiliations:** Department of Cellular Pathology, Blackpool Teaching Hospitals NHS Foundation Trust, Whinney Heys Road, Blackpool FY3 8NR, UK; francis.martin2@nhs.net

As healthcare tools increasingly move towards a more digital and computational format, there is an increasing need for sensor-based technologies that allow for rapid screening and/or diagnostics [1]. Biospectroscopy is an emerging interdisciplinary systems biology approach with the potential to revolutionise the management and triage of patients in point-of-care settings [2]. The notion of a testing platform that is reagent-free and requires minimal sample preparation is incredibly attractive, especially in the context of achieving a cost-effective yet efficient analysis of numerous samples or patients in routine clinical practices [3]. Data fusion, also known as data blocking, combines two or more systems biology datasets from different “omics” modalities to generate greater organism insights and can be exploited to provide an even more integrated approach [4]. Biospectroscopy approaches employ spectrochemical technologies (Table 1) primarily associated with mid-infrared (MIR) or Raman platforms [5], although near-infrared (NIR) can also be included [4]. The application of spectrochemical methods, whose output in the form of vibrational spectra is fed into chemometric algorithms, is gaining increasing recognition as potential screening and/or diagnostic tools in clinical settings [6]. These methods have been applied to point spectra acquisition from biological samples (i.e., obtained during typical clinical practice) for classification or feature extraction [7] right through to hyperspectral imaging [8]. It is a new paradigm that can be exploited to interrogate cytology [9], tissues [10], or biofluids [11] in order to deliver a new intervention strategy [12] for screening and/or diagnosis.

There is now a large body of extensive studies examining the applicability and efficacy of biospectroscopy techniques in potential healthcare settings; these have already been applied to multiple endpoints [1], including investigations into cervical cytology [13], the prostate [14], neurodegenerative disease [15], neuro-oncology [16], and virology [17]. At every juncture, the application of such an approach requires a detailed inter-disciplinary cross-talk between professionals in very different fields. The first objective of this exchange is to determine whether the problem being addressed is relevant in a medical context [18]. Subsequently, it is necessary to ascertain whether a relevant sample can be obtained and prepared in a fashion that effectively accommodates the underlying physics of spectrochemical analysis, ensuring a favourable signal-to-noise ratio (SNR) [1]. Finally, it is crucial to evaluate whether the spectral output is large and robust enough, despite its complexity, to avoid issues such as over-fitting when the data (often highly complex) is inputted into powerful computational algorithms [19]. If each step is not adequately addressed, the result is essentially meaningless. First, the scientist needs to ascertain from the medical professional the exact problem within a chosen area and determine that the latter encounters this in their day-to-day clinical setting. It is surprisingly easy for the scientist to take a naïve view of what is truly clinically relevant. For instance, every woman in the Western world will likely be infected by human papillomavirus (HPV), especially in the second and third decades of life; of greater clinical relevance are those whose immune system fails to clear the HPV infection by their fourth decade [18]. Following on, the scientist needs to fully understand the biological constraints and marry these to the physics so as to be able to derive optimum spectral SNR. Consequently, the sample will need to be thick enough [19] and on the appropriate substrate [11,20,21]. The over-arching importance at this juncture is to understand a typical care setting and the throughput of samples. Complex sample preparation methodologies using expensive reagents or substrates are not amenable to the economic constraints of a laboratory that needs to handle multiple thousands or more samples per day. Finally, the output needs to be robust and consistent in quality, with minimal numbers of outliers. In the case of biospectroscopy in a clinical setting, it is a spectral output, and this is inputted into a predictive algorithm that then gives a readout, which may be in the form of a traffic light indicator [22]. Such computational algorithms need to be capable of delivering high sensitivity and specificity and facilitating standardisation across multiple settings [23]. The exciting aspect of this is that such computational algorithms that readily lead to machine learning and artificial intelligence techniques are readily automated for the end-user.

Despite this apparent methodological complexity, the emerging evidence is that biospectroscopy techniques have a potential role in healthcare settings because, ultimately, for the end-user, their application is more about technical consistency and less about a deep understanding of the aforementioned individual steps. Technical consistency, in this case, refers to the standardisation of sample preparation and consistent delivery to the sensor, something which standard operating procedures can be readily developed for and which healthcare systems use already in routine practice. A major driver in the push towards the implementation of biospectroscopy techniques in healthcare is the increasing processing power of desktop computers (or even mobile phones) that allow for the rapid harnessing of sophisticated computational algorithms and the developing usage of databases in an increasingly digital era; this means that there is an increasing acceptance amongst healthcare professionals of remote algorithm-based outputs on which clinical decisions, such as patient triage or diagnosis, can be made. In combination with its general applicability to a range of screening and/or diagnostic scenarios (e.g., on cytology, tissue sections, or liquid biopsies), one would envisage that biospectroscopy platforms could be readily embedded into healthcare point-of-care screening and/or diagnostics settings at a cost consideration not dissimilar to current practice; this is a critically important point. For instance, pathology laboratories use glass slides because they are cheap, easy to use, and robust, thus allowing a typical laboratory to process hundreds to thousands of in-house samples per day. A replacement approach should not necessitate the inclusion of an expensive component such as gold-coated slides (maybe >100 times the price compared to ordinary slides) or fragile materials (e.g., BaF_2_ windows). Increasingly, from being an academic laboratory-based tool, biospectroscopy has evolved to facilitate clinical translation. For initial screenings of large numbers of patients, this is important. This initial biospectroscopic triage would then allow the targeting of subsets of queried patients with more detailed analyses (e.g., scans and mutation analysis).

The possibilities raised by the emergence of biospectroscopy techniques extend from the acquisition and processing of point spectra to the construction of images of an interrogated biological sample (e.g., biopsy ex vivo or tissue section). While the acquisition of point spectra allows one to determine the classification of normal versus variant (e.g., disease) or predictive analysis for screening and/or diagnosis, the use of spectral maps allows one to construct an image of the cell and/or tissue architecture [24,25] or even a profile map of the bio-distribution of a particular entity, such as a biomolecule [26]; this lends itself to an exciting new horizon that could provide an alternative to traditional dye-based histopathology. Traditionally, one could have looked at what seemed a normal tissue architecture based on different coloured dyes binding to various cellular entities with no indication that there may be insidious disease about to emerge; the reason being that in this format, no underlying biochemical information is discernible [27]. The paradigm shift in biospectroscopy is that the spectrum-derived image map, in addition to showing tissue architecture, also contains and exhibits underlying biochemical information [28]. Such capability to be integrated into clinical workflows [29] could facilitate the objective prediction of disease [30] and even response to treatment [31], e.g., platinum-based chemotherapy. To further advance the combination of spectroscopy and imaging, hyperspectral techniques can be employed. Herein, for the image segment generated, each image position (i.e., pixel) generates a spectrum, thus creating a three-dimensional (3-D) object for each measurement; this is known as a hyperspectral “data-cube”. The value of this approach lies in its ability to enhance the acquired data by providing access to spatial information along the x- and y-axes, as well as chemical information along the *z*-axis. One can visualise this by stacking up single-segment images at different wavelengths. As mentioned above, computational approaches primarily based on multivariate analysis are required for data processing and interpretation [32]. Again, the potential of such analyses is that they allow feature extraction towards classification or longitudinal temporal predictive response with minimal input interference, a format that lends itself to the reliable creation of computational models to analyse images in an automated fashion. One could surmise that biospectroscopy, with this goal in mind, has many potential healthcare applications.

An area where biospectroscopy techniques have already made significant inroads is in the application (primarily of Raman spectroscopy) of real-time intra-operative assessment of tumour margins [33,34,35]. Because of the potential for fast, essentially real-time, acquisition [36] that relies on the scattering of monochromatic light, which interacts with molecular vibrations, affecting up-shifting or down-shifting in the energy of photons, the implementation of this platform, thus, provides an objective, non-destructive and fast intra-operative assessment of a resection surface (including deep soft tissue layers) [37]. Given its relative immunity to water interference (an important consideration for in vivo applications), Raman spectroscopy is well suited for surgical purposes. There is growing evidence of its safe and effective usage in humans in areas including neurosurgery, whereby intra-operative Raman fibre optic probes in combination with brain biopsy probes [38,39] are being developed. This approach not only enables the prompt determination of whether a sample is classified as tumour or non-tumour, but it also facilitates the simultaneous generation of outputs derived from high SNR spectra and genomic/molecular/biochemical information. Thus, using classification models, excised brain tissue can be immediately genotyped according to the derived Raman spectrum; for instance, it was shown possible to accurately and rapidly classify gliomas according to isocitrate dehydrogenase mutations and 1p/19q deletions [40]. As personalised medicine is increasingly applied to manage individual patients, this approach shows significant promise. An obvious step from the implementation of a spectrochemical intra-operative probe would be to replace or combine a digital readout with an acoustic indicator, which the surgeon could hear as they operate without being distracted.

Although much work applying and investigating the usefulness of biospectroscopy has been undertaken in the oncology field, the methodology has also been successfully studied in many other areas, including cardiology [41], infectious diseases [42,43], amyloid pathologies [44], fibromyalgia syndrome [45], and mood disorders [46]. There is now a comprehensive body of literature containing multiple studies investigating and validating different aspects of this approach. Given the digital framework of biospectroscopy approaches in that the clinical output is computational and data-driven, the regulatory framework governing its usage in healthcare may need to differ from more traditional laboratory-based methods dependent on genomic or proteomic readouts. However, it is a relatively easy prediction to suggest that digital platforms based on biospectroscopy techniques may revolutionise healthcare screening and/or diagnostics in a form not dissimilar to a Star Trek-style medical tricorder, whereby a rapid scan allows the practitioner to quickly gather data on a patient and instantly work out what is wrong with them.

## Figures and Tables

**Table 1:** Typical spectrochemical techniques used in biospectroscopy studies.

Sensor	Spectral Range
Near-infrared spectroscopy	12,820–4000 cm^−1^
Attenuated total reflection Fourier-transform (ATR-FTIR) spectroscopy	4000–400 cm^−1^ (mid-IR)
Fourier-transform infrared microspectroscopy	4000–400 cm^−1^ (mid-IR)
Hyperspectral imaging	4000–400 cm^−1^ (mid-IR)
Raman spectroscopy	4000–50 cm^−1^
Fluorescence spectroscopy	796–1054 nm
Terahertz (THz) spectroscopy	0.03–3 mm
Optical photothermal infrared microscopy	4000–400 cm^−1^ (mid-IR)
